# Does resuscitation status affect decision making in a deteriorating patient? Results from a randomised vignette study

**DOI:** 10.1111/jep.12559

**Published:** 2016-05-30

**Authors:** Suzanne Moffat, Jane Skinner, Zoë Fritz

**Affiliations:** ^1^ Health SciencesUniversity of East AngliaNorwichNorfolkUK; ^2^ Department of MedicineUniversity of East AngliaNorwichNorfolkUK; ^3^ Acute MedicineCambridge University Hospitals NHS Foundation TrustCambridgeCambridgeshireUK

**Keywords:** clinical safety, healthcare, medical education

## Abstract

**Aims and objectives:**

The aim of this paper is to determine the influence of do not attempt cardiopulmonary resuscitation (DNACPR) orders and the Universal Form of Treatment Options (‘UFTO’: an alternative approach that contextualizes the resuscitation decision within an overall treatment plan) on nurses' decision making about a deteriorating patient.

**Methods:**

An online survey with a developing case scenario across three timeframes was used on 231 nurses from 10 National Health Service Trusts. Nurses were randomised into three groups: DNACPR, the UFTO and no‐form. Statements were pooled into four subcategories: Increasing Monitoring, Escalating Concern, Initiating Treatments and Comfort Measures.

**Results:**

Reported decisions were different across the three groups. Nurses in the DNACPR group agreed or strongly agreed to initiate fewer intense nursing interventions than the UFTO and no‐form groups (*P* < 0.001) overall and across subcategories of Increase Monitoring, Escalate Concern and Initiate Treatments (all *P* < 0.001). There was no difference between the UFTO and no‐form groups overall (*P* = 0.795) or in the subcategories. No difference in Comfort Measures were observed (*P* = 0.201) between the three groups.

**Conclusion:**

The presence of a DNACPR order appears to influence nurse decision making in a deteriorating patient vignette. Differences were not observed in the UFTO and no‐form group. The UFTO may improve the way nurses modulate their behaviours towards critically ill patients with DNACPR status. More hospitals should consider adopting an approach where the resuscitation decisions are contextualised within overall goals of care.

## Introduction

In the United Kingdom (UK) cardiopulmonary resuscitation (CPR) is attempted for a quarter of the 285 000 patients who die in an acute hospital setting each year 1. If attempted CPR is not desired – because of patient request or because it is unlikely to be successful – then this is documented on a do not attempt cardiopulmonary resuscitation (DNACPR) order. A DNACPR order is precise and narrow, a written decision that applies only to CPR with all other treatment and care for that patient continuing 2. Yet a review of the literature suggests DNACPR orders are associated with many problems including the following: variability in triggers and influences; variability in implementation of documentation; misunderstanding that DNACPR always means that patients are approaching the end‐of‐life 3; misunderstanding that DNACPR means that other treatments should be withheld; and evidence that patients with DNACPR orders receive fewer appropriate treatments than those without them 4, 5, 6.

This last point is particularly relevant for the ‘deteriorating patient’: in the hours leading to cardiac arrest, 84% of patients show signs of physiological instability 7. Early recognition and response to physiological deterioration can dramatically reduce cardiac arrests 8. Nurses at the bedside are most likely to recognise deterioration and initiate decisions on how to manage, treat and escalate concern. However, DNACPR orders are associated with nurses delivering less aggressive therapies 9, 10, 11, reduced levels of monitoring activity 11, 12, 13 and less urgent escalation to senior support 14, 15. In contrast, comfort measures in patients with DNACPR orders increase or remain unchanged 10, 16, 17.

One potential solution to ensure timely plans are in place about CPR and other medical decisions has been the introduction of overall treatment plans 18, 19. These focus on what interventions a patient will or will not receive, including CPR, in the event of deterioration. Thirteen per cent of UK hospitals already incorporate DNACPR status within an escalation of care plan 20. One example in the UK is the Universal Form for Treatment Options (UFTO). In contrast with the DNACPR form, which focuses on one treatment (CPR) to be withheld, the UFTO establishes overall goals of care and identifies which treatments, including CPR, should or should not be given. In a mixed method before‐and‐after study with contemporaneous case controls, the introduction of the UFTO was associated with a significant reduction (*P* < 0.001) in harm events (as measured by the global trigger tool) in those patients whom a DNACPR decision had been made 21. It was observed that nurses stopped labelling patients by their resuscitation status and instead talked about what other treatments should be considered.

Although a number of studies have determined the isolated effects of the DNACPR order 13, 22, 23, 24, little is known of the isolated effects of the UFTO or any similar initiatives. Randomised vignette surveys are useful in evaluating reported behaviours in health care professionals 25, 26, 27, 28, 29. We designed this experimentally controlled survey to assess if the same information presented on a different form could affect how nurses respond to a hypothetical deteriorating patient.

## Methods

### Questionnaire

An online survey was developed through an iterative process with advice from an expert group consisting of a medical consultant and four specialist nurses. A case scenario was created of a 75‐year‐old lady with a newly diagnosed cancer with good functional status who developed severe sepsis. Key points in a deterioration pathway where the nurse is responsible or can influence outcome were identified 8, 30. Forty five sequential statements across three time frames over 9 hours were based upon these key points, with associated questions asking how strongly the participant agreed with each statement.

The statements addressed the following areas:

- *Escalating Concern*. Actions directed towards informing senior support of the patient's deterioration.
- *Increasing Monitoring*. Aimed at monitoring the patient's condition.
- *Initiating Treatments*. Aimed at stopping or reversing the presenting problem, sign or symptom.
- *Comfort Measures*. Actions directed at minimising discomfort from symptoms without direct attention to the underlying disease 23.

The survey was pilot tested on 15 eligible nurses who were subsequently removed from the population frame. Positive feedback was received regarding readability, relevance of content and layout while several changes were made to improve clarity and language including removing two items. The time to complete the survey was reported to be approximately 10 minutes.

The primary outcome measure was the sum of the nurse's believed agreement of initiating interventions. Strength of agreement was coded on an ordinal scale ranging from ‘strongly agree’ (1) to ‘strongly disagree’ (4). Approximately a quarter of the statements were worded in the negative and subsequently reversed for analysis. The secondary outcome was the sum of the nurse's 1–4 scale responses for each of the four subcategories between groups: Escalating Concerns (11 items), Increasing Monitoring (9 items), Initiating Treatments (14 items) and Comfort Measures (9 items). The variables were approximately normally distributed.

Analysis using one‐way ANOVA was carried out to test for differences in group means where the assumption of homogeneity was met using Levene's test; Welch's adjusted F‐test was used where it was not. If a significant difference was found in group means, Tukey's honestly significant difference *post‐hoc* analysis was performed to make the three possible pairwise comparisons between group means, where the variances could be assumed to be equal; Games‐Howell was used where they could not. Statistical significance was set at *P* < 0.05.

The responses to 41 individual statements were measured using a 1–4 category Likert Scale. The 4‐point Likert scale was combined into two scales (disagree/strongly disagree and agree/strongly agree) for ease of presentation and interpretation, and the answers presented as percentages. Demographic questions were placed at the beginning of the questionnaire.

**Figure 1:**
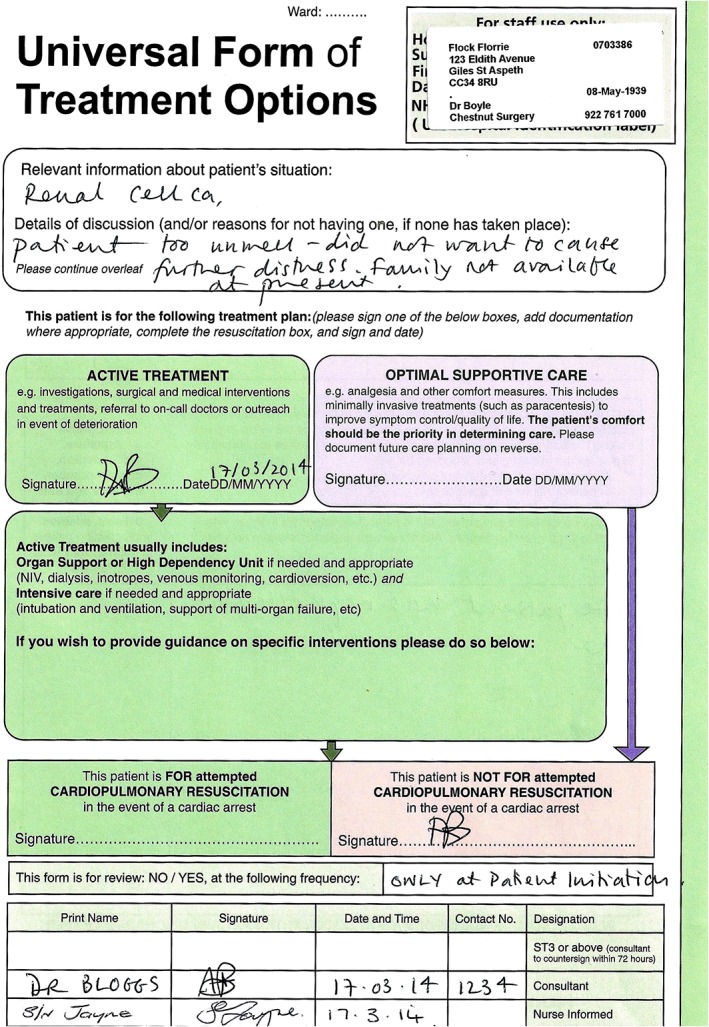
Completed Universal Form of Treatment Options as seen by participants in the Universal Form of Treatment Options group.

**Figure 2:**
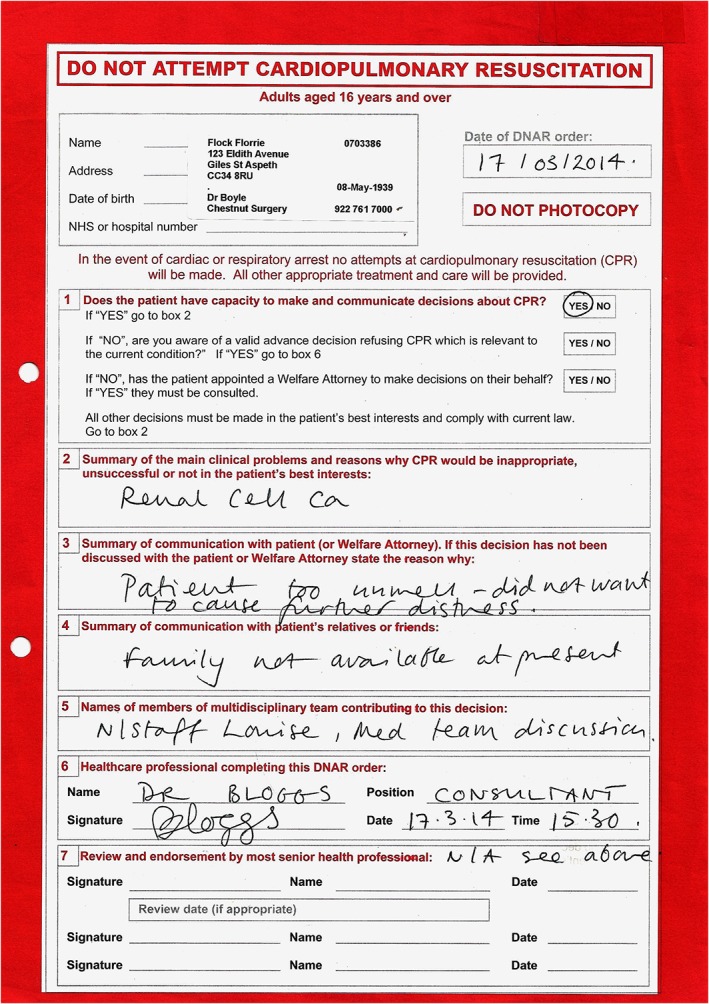
Completed do not attempt cardiopulmonary resuscitation order as seen by participants in the do not attempt cardiopulmonary resuscitation group.

Three versions of the survey were simultaneously created: one with a DNACPR order, one with UFTO and one with no form. The UFTO (Fig. 1) and DNACPR order (Fig. 2) contained the same instruction except the UFTO had the additional box ‘for active treatment’ signed. The DNACPR order was written on a standard Resuscitation Council (UK) DNACPR proforma that is recognised as standard documentation. Questionnaire design and formatting, including the use of ‘branching’ to create randomization, was facilitated by the online provider FluidSurvey™ (www.fluidsurvey.com).

### Statistical analysis

Data were analysed using SPSS v21.0 (SPSS v21.0, IBM, New York, USA). A total of 231 eligible questionnaires were received. Twenty nine missed fewer than 50% of questions; the mode of answers from other participants was used for the missing questions. A confirmatory analysis including only the 202 with complete answers was also carried out.

Demographic characteristics were similar among the three groups (Table 1). Most respondents were female, experienced nurses.

**Table 1:** Baseline demographic characteristics of nurses who participated in the survey

	The UFTO (*n* = 74)	DNACPR (*n* = 68)	No‐form (*n* = 89)	Total (*n* = 231)
Age				
21–30	14	9	17	40
31–40	21	20	19	78
41–50	24	24	37	85
50+	15	15	16	46
Gender				
Male	13	11	9	33
Female	61	57	80	198
Number of years nursing				
<1	4	1	4	9
1–2	4	7	7	18
3–4	10	5	9	24
5–10	11	6	15	32
11–20	21	20	20	61
>20	24	29	34	87
Frequency of caring for DNACPR patients				
Never	2	6	2	10
Not in past year	12	12	21	45
<2 a month	33	17	38	88
>2 a month	27	33	28	88

DNACPR, do not attempt cardiopulmonary resuscitation; UFTP, Universal Form of Treatment Options.

### Study approval, consent and data collection

Ethics approval was obtained from the University of East Anglia Research Ethics Committee. Research Governance was obtained from each participating organisation.

G‐Power analysis 31 with settings for a fixed effects one‐way ANOVA was used to calculate the sample size for between‐group analyses of the primary outcome measure (intense nursing interventions). Power was set at 0.9, significance level was set at 0.05 and effect size (Cohen's f) was set at 0.25 (medium) 32 for the three‐group design. It was calculated that a sample size of 207 was needed (69 participants in each group).

Eligible participants were band 5 and 6 nurses who had attended the Resuscitation Council (UK) Immediate Life Support course in the previous 12 months. Participants across 10 NHS Trusts were included to ensure a range of hospitals across England and Wales. The surveys were distributed via the local hospital resuscitation officer to maintain confidentiality and optimise response rate. A letter of invitation was emailed along with a link to the survey; participants were informed that the study was evaluating the effect of clinical judgement on the nursing care of a patient. No specific mention of resuscitation was mentioned to minimise response bias. Consent to participate was implicit by completion of a returned survey. The survey was completed anonymously.

## Results

The resuscitation officers sent 2167 emails with links to questionnaires; we subsequently discovered many of these were not accessible to the recipients because of hospital firewalls. Because of this, it was not possible to determine an accurate response rate to the email. Four hundred forty‐six people clicked on the survey; 202 answered all questions (45% completion rate).

### Relationship between the groups comparing intense nursing interventions

Welch's adjusted F‐test revealed significant differences between the groups (*P* < 0.001). The Games‐Howell test showed the DNACPR group scores were significantly higher than the no‐form group and the UFTO group (Table 2), suggesting the nurses were more likely to withhold interventions when a DNACPR order was in place than when there was no form or an UFTO was in place. Results were similar when only the 202 fully completed questionnaires were included.

**Table 2:** Results for all groups comparing intense nursing interventions

	The UFTO (*n* = 74)	DNACPR (*n* = 68)	No‐form (*n* = 89)	Total (*n* = 231)	Test of equality of means				Post hoc tests		
									UFTO vs. no‐form	DNACPR vs. UFTO	No‐form vs. DNACPR
	Mean SD	Mean SD	Mean SD	Mean SD	Statistic	df1	df2	*p* value	*p* value	*p* value	*p* value
Intense nursing interventions	59.3	66.7	58.3	61.1	10.19*	2	140.17	<0.001	0.795†	0.001†	<0.001†
	9.8	13.2	9.3	11.3							

* Welch's adjusted F‐ratio, asymptotically F distributed.

† Games‐Howell pairwise comparison.

UFTO, Universal Form of Treatment Options; DNACPR, do not attempt cardiopulmonary resuscitation; SD, standard deviation.

### Relationship between the groups comparing Escalating Concern, Initiating Treatments, Increasing Observations and Comfort Measures

There were significant differences between the groups in the subcategories relating to intensity – Escalating Concern, Initiating Treatment and Increasing Monitoring (all *P* ≤ 0.001) (Table 3). In the Comfort Measures category, there were no significant differences (*P* = 0.201) between groups. *Post hoc* tests showed the DNACPR group was significantly different to the no‐form (*P* < 0.001) and the UFTO (*P* = 0.001) groups in the Escalating Concern and Increasing Monitoring categories. In the Initiating Treatments sub‐category, the DNACPR group was significantly different to the no‐form (*P* < 0.001) and the UFTO (*P* = 0.024) group. There were no differences observed between the UFTO and no‐form in these three subcategories aimed towards cure. Results were similar for the 202 fully completed questionnaires alone, except that the pairwise comparison of DNACPR versus UFTO was no longer significant for initiating treatment (*P* = 0.058) and increasing observations (*P* = 0.053).

**Table 3:** Results for all groups comparing subcategories of nursing interventions

	The UFTO (*n* = 74)	DNACPR (*n* = 68)	No‐form (*n* = 89)	Total (*n* = 231)	Test of equality of means				Post hoc tests		
									UFTO vs. no‐form	DNACPR vs. UFTO	No‐form vs. DNACPR
	Mean SD	Mean SD	Mean SD	Mean SD	Statistic	df1	df2	*p* value	*p* value	*p* value	*p* value
Escalating Concern	17.6	20.6	17.9	18.7	10.21*	2	142.30	<0.001	0.828†	<0.001†	<0.001†
	3.6	4.9	3.7	3.6
Initiating Treatment	27.7	29.7	26.8	28.0	8.57‡	2	228	0.001	0.349§	0.024§	<0.001§
	4.3	4.7	4.5	4.6
Increasing Monitoring	13.6	16.2	13.2	14.9	7.18*	2	136.87	0.001	0.811†	0.007†	0.001†
	3.6	4.9	3.1	3.6
Comfort Measures	14.6	14.9	15.4	15.0	1.54‡	2	228	0.201	
	3.0	3.3	3.1	3.1

* Welch's adjusted F‐ratio, asymptotically F distributed.

† Games‐Howell pairwise comparison.

‡ One‐way ANOVA.

§ Tukey's Honestly Significant Difference pairwise comparison.

UFTO, Universal Form of Treatment Options; DNACPR, do not attempt cardiopulmonary resuscitation; SD, standard deviation.

### Relationship between the groups and cardiopulmonary resuscitation practices

The four additional items relating to CPR practices were presented separately (Table 4). Nurses were asked whether they would start CPR if the patient became pulseless. A significant disparity between nurses in the DNACPR group (89%) and nurses in the UFTO group (45%) was observed, while approximately one fifth of the no‐form group disagreed that they would start CPR. Both the UFTO and DNACPR groups gave split views about commencing bag‐valve‐mask ventilations when the respiratory rate fell below normal limits of eight breaths per minute with half agreeing bag‐valve‐mask ventilation should be commenced (49% vs. 49%),while 78% in the no‐form group would do so. A statement relating to nurses' personal opinion asked whether they would *want* to start chest compressions if the patient became pulseless: over a third (38%) of the no‐form group would not want to start chest compressions when no CPR decision had been made. This rose to 58% in the UFTO group and 82% in the DNACPR group. Finally, 17% of the DNACPR group, 49% of the UFTO group and 75% of the no‐form group agreed the patient should be defibrillated if they went into a shockable cardiac arrest rhythm.

**Table 4:** Number of cardiopulmonary resuscitation related treatment interventions nurses agreed/strongly agreed to initiate in the presence of the UFTO or DNACPR order or neither

	The UFTO (*n* = 74) %	DNACPR (*n* = 68) %	No‐form (*n* = 89) %
‘If Mrs F became pulseless I would start CPR’	45	11	79
‘Bag‐valve‐mask ventilations should be started if the respiratory rate decreases below 8’	49	49	78
‘I would want to start CPR if Mrs F had a cardiac arrest’	42	18	62
‘Mrs F should be defibrillated if she goes into cardiac arrest in a shockable rhythm.’	49	17	75

CPR, cardiopulmonary resuscitation; UFTO, Universal Form of Treatment Options; DNACPR, do not attempt cardiopulmonary resuscitation.

## Discussion

In this deteriorating patient vignette study, nurses initiated fewer interventions beyond CPR in those patients with a DNACPR order than in those with no‐form and the UFTO; these differences were not observed between the UFTO and no‐form groups. The results of this study are encouraging in that the UFTO appears to have succeeded in ensuring that the ‘do not resuscitate’ decision did not undermine other treatment decisions: the patients with UFTO were treated similarly to those with no order. The UFTO may improve the way nurses modulate their behaviours towards critically ill DNACPR patients, and more centres should consider adopting an approach where the resuscitation decisions is contextualised within overall goals of care.

Previous clinical studies had demonstrated that those with DNACPR orders received fewer interventions 33 and observations 4, 5, 6, 12, but these studies had been criticised although they accounted for all measurable variables (age, comorbidity, etc.). It was suggested that the DNACPR order conveys something about prognosis that is not measurable – the apocryphal ‘end‐of‐the‐bed‐o‐gram’ that allows doctors and nurses to prognosticate outside known statistics and that this explained the poor outcomes observed in patients with DNACPR orders. The vignette studies of Henneman 13 and Beach 22 had a more sinister implication: they indicated that nurses and doctors gave patients with a DNACPR order fewer treatments *because* they had a DNACPR order. Although DNACPR status is not intended to alter patients care in any way beyond withholding CPR, it was being interpreted to mean that other care should also be withheld. It was in part because of these studies that the UFTO was developed: to ensure a delineation between the resuscitation decision and the overall goals of care, allowing clinicians to document that a patient was for other active treatments while not being for resuscitation in the event of a cardiac arrest.

The experimental vignette design removes all confounders and thus provides valuable insight into the isolated effect of the UFTO compared with a DNACPR order on nurse‐led or nurse‐initiated interventions. The results show significant differences between nurses in the DNACPR group compared with nurses in the UFTO and no‐form groups: nurses carried out fewer interventions on a patient with a DNACPR order than on those patients with no‐form in the Initiate Treatments, Escalate Concern and Increase Monitoring subcategories; the UFTO group was treated analogously to the no‐form group. It would have been appropriate for this patient to be treated in compliance with the Surviving Sepsis resuscitation protocol 34; yet the DNACPR group believed fewer interventions were necessary. Even if the nurse cannot prescribe drugs or perform invasive procedures, they can omit treatments pending medical review or escalate their concerns and press for admission to a higher dependency setting. Consistent with the findings of others 12, 17, our results report nurses would deliver the same level of comfort measures across all groups. It seems that DNACPR status is not a marker to give less care altogether, just to reduce those elements of care which might prevent further deterioration.

The reasons for the differences between the DNACPR and other groups need further exploration; it is possible that DNACPR was interpreted as an indication that the patient was approaching end‐of‐life and excessive monitoring and therapies could cause excessive discomfort. In fact, around 50% of patients with DNACPR orders are discharged home, and over a third are alive at a year 35. An alternative interpretation is that the nurses did not understand the UFTO and believed that the patients remained for all treatments, as with the no‐form group, or that they believed that the UFTO gave them more leeway in deciding which elements of resuscitation might be appropriate.

General nurses are an important and often neglected group when it comes to understanding patients with DNACPR status; this survey across 10 Trusts gives considerable insight into how they interpret intensity of care in a patient with DNACPR status using different forms. The participating nurses had considerable experience with DNACPR status patients, yet there was still significant misunderstanding about what the order means and how to manage deteriorating patients with such orders. Hennemen and Beach's studies were carried out 10 years ago, and it is disheartening to see so little change in attitudes, particularly because vignette studies are likely to over‐estimate what nurses would do in real life.

The split results regarding the course of management for the decreasing respiratory rate in the scenario reveals uncertainty in how to actively manage deterioration in a patient with DNACPR status. The UK guidelines state ‘A DNACPR decision does not override clinical judgement in the unlikely event of a reversible cause of the person's respiratory or cardiac arrest that does not match the circumstances envisaged when that decision was made and recorded’^2^. Yet in an emergency, interpreting a falling respiratory rate within ‘matched circumstances’ might not be straightforward. This highlights the difficulty faced by front line staff when a plan is not clearly defined.

The final set of questions – on practices closely related to, or part of CPR – did not address any of our initial aims. We included it in order to reveal misunderstandings about which elements of practice are included in ‘CPR’. Interpretation of this section has its own limitations; however, we feel it important to point out that more elements of resuscitation were carried out in the UFTO than the DNACPR group. Forty five per cent of the UFTO group said they would start chest compressions, and almost half agreed the patient should be defibrillated if the patient went into a shockable rhythm, while just under a fifth of the DNACPR order group agreed to start CPR and defibrillate the patient in the event of a cardiac arrest. These are interesting findings in light of the NCEPOD report 36, which revealed 52 of the 552 patients in whom resuscitation was attempted had a DNACPR order, suggesting DNACPR orders may not be followed around 10% of the time. This may reflect the ambiguities we all see in deteriorating patients: defibrillation can be provided in ventricular tachycardia, and a bag‐valve‐mask can be provided as an airway adjunct. Although data was not collected to answer why these procedures should be started, possible explanations include that nurses do not consider defibrillation and manual ventilation the same as CPR – just other active treatments – or they may be waiting for a doctor to arrive and confirm what they should do. While our results suggest that the nuances of the UFTO might be missing, within the UFTO trial 21 there were no inappropriate resuscitation attempts, suggesting in clinical practice UFTO is not misunderstood to mean that CPR should still be given.

Limitations of our study include the accuracy in determining the response rate; some hospitals had firewall protection preventing nurses accessing the questionnaire at work and several resuscitation officers reported emails had been returned. Future studies should take account of this problem when targeting NHS staff, and consider a response rate of those surveys opened, rather than all emails sent. The difficulties with vignette studies in general are the lack of further instruction and information is processed less carefully and efficiently than under real conditions 37. As there was no education on UFTO, and it was not a habituated practice, nurses may have been focusing on the active treatment element rather than the DNACPR order; questions relating explicitly to whether UFTO was understood may have been helpful in exploring the reasons for the answers regarding CPR practices. To avoid confusion about which elements of treatment are included in resuscitation, we suggest that resuscitation training courses should include scenarios that incorporate these elements when a DNACPR status patient is deteriorating.

The DNACPR order is still misunderstood and could be associated with nurses initiating fewer interventions. This is despite attempts over the years to address this problem, not least with lengthening the acronym from DNR (do not attempt resuscitation) to DNAR (do not attempt resuscitation) to DNACPR to improve the clarity of the instruction (although all the acronyms are still in common parlance.) Attempts at education do not seem to have had long‐term effects 3. Ethnographic evidence suggests that some of the problem may be the subliminal messaging in the red form at the front of the notes or in the primacy of the ‘DNACPR’ label in nursing handover 38; a negative label focussing on a treatment to be withheld appears to easily have unintended consequences. Contextualising the resuscitation decision within overall goals of care and emphasising which treatments should be given may help nurses and doctors focus on giving the appropriate treatments. In the UK, the Resuscitation Council and the Royal College of Nurses have chaired a group to create a national approach to resuscitation decisions that does this, drawing on evidence from UFTO and other approaches 39. While this will need further evaluation, it is heartening to see that a change in culture is taking place.

## Conflicts of interest

We, the authors, state that we do not have any financial and/or personal relationships with other people or organisations that could inappropriately influence (bias) our work.

## Supporting information

Clinical setting and history.

Supporting info item
