# Supplementary material for: Does resuscitation status affect decision making in a deteriorating patient? Results from a randomised vignette study
Source: J Eval Clin Pract. 2016 May 30;22(6):917–23. doi: 10.1111/jep.12559 (PMC5111586; doi:10.1111/jep.12559)
Supplement: Supplementary file 1 — Supporting info item [file JEP-22-917-s001.docx]

**Supporting Information**

**Part 1.**

**Clinical setting and history.**

**Situation - 15.00hrs.** Mrs F is a 75 year old female. She has been admitted to hospital with Atrial Fibrillation (AF), abdominal pain and pain when passing urine. Computed Axial Tomography (CT) scan reveals a renal mass with bleeding into it. Treatment with oral Digoxin and oral broad spectrum antibiotics are started. She has been referred to the Palliative Care Team.

**Background.** She has unintentially lost 10 kilograms in weight in the past 6 months. Lives alone with daily help. Uses a stick to walk. Mrs F has requested a Chaplain visit during her admission.

Previous history of hypertension and a small stroke 10 years ago.

Next of kin is her daughter who lives 200 miles away

**Assessment.** Over the first 24 hours her Respiratory Rate has been 24, heart rate 125bpm, Blood pressure 115/ 60mmHg, temp 37.9 and O2 Saturations 94%. National Early Warning Score (NEWS) has remained at 5 since the ward round 3 hours previously. Observations are being performed 4 hourly. She appears quiet and withdrawn.

What would your **Recommendations** be**?**

***Outreach Team = nurse led escalation team.**

**** FY1 = Foundation Year 1 doctor.**

**Click on the link to see the form (if randomised to the UFTO or DNACPR order). To get back to the scenario click the ‘back arrow’ at the top left corner of the page.**

| **INTERVENTIONS.** | | | | | |
| --- | --- | --- | --- | --- | --- |
|  | | Strongly  agree | Agree | Disagree | Strongly disagree |
| 1. | I would increase the observations | □ | □ | □ | □ |
| 2. | I would commence a fluid balance chart | □ | □ | □ | □ |
| 3. | I would move her to a bed near the nurses station | □ | □ | □ | □ |
| 4. | I would contact the Outreach Team* for review | □ | □ | □ | □ |
| 5. | I would contact the FY1** | □ | □ | □ | □ |
| 6. | If a Registrar was not involved I would not contact them | □ | □ | □ | □ |
| 7. | I would not phone the family to inform them of the patient’s condition | □ | □ | □ | □ |
| 8. | I would contact the Chaplain | □ | □ | □ | □ |
| 9. | I would contact the Palliative Care Team again to review. | □ | □ | □ | □ |
| 10. | I would not administer oxygen until it had been prescribed by a doctor or the Outreach Team | □ | □ | □ | □ |
| 11. | I would offer Mrs F the opportunity to talk about her concerns | □ | □ | □ | □ |
| 12. | My priority is ensuring comfort rather than recording observations | □ | □ | □ | □ |
| 13. | I would request fluids are commenced | □ | □ | □ | □ |
| 14. | I would not consider blood cultures are a priority | □ | □ | □ | □ |
| 15. | I would request the antibiotics are changed from oral to IV route | □ | □ | □ | □ |
| 16. | A general ward is appropriate for Mrs F | □ | □ | □ | □ |
| 17. | I would catheterise her | □ | □ | □ | □ |

**Part 2.**

**Situation - 19.30hrs.** After review by the FY1 Mrs F is commenced on 4 litres of Oxygen via nasal cannula and started on a normal saline intravenous (IV) infusion 500ml over 4 hours. The Outreach Team have been informed but they are busy with other patients and will come as soon as possible. The infusion is alarming as the cannula has infiltrated.

**Background.** Abdominal pain caused by renal mass. In AF. Referred to Palliative Care Team.

**Assessment.** Respiratory Rate 28, Heart Rate 129 bpm, Blood Pressure 115/55mmHg, Temp 38.2 and o2 Saturations 94%. She has been asleep most of the afternoon, waking occasionally with abdominal discomfort. Urine output 50mls over 4 hours.

What would your **Recommendations** be**?**

**Click on the link to see the form (if randomised to the UFTO or DNACPR order). To get back to the scenario click the ‘back arrow’ at the top left corner of the page.**

| **INTERVENTIONS.** | | | | | |
| --- | --- | --- | --- | --- | --- |
|  | | Strongly  agree | Agree | Disagree | Strongly disagree |
| 1. | I would wake Mrs F to encourage oral fluids | □ | □ | □ | □ |
| 2. | I would move Mrs F to a bed near the nurses’ station if not already | □ | □ | □ | □ |
| 3. | I would not increase the frequency of her observations | □ | □ | □ | □ |
| 4. | I would ensure a fluid balance chart is in place if not already | □ | □ | □ | □ |
| 5. | I would record a Glasgow Coma Score or AVPU | □ | □ | □ | □ |
| 6. | I would contact the Outreach Team or FY1 again | □ | □ | □ | □ |
| 7. | I would wait for 45 minutes to bleep the FY1 or Outreach Team again if I didn’t get an initial response | □ | □ | □ | □ |
| 8. | I would contact the Registrar if the FY1 was not going to contact them | □ | □ | □ | □ |
| 9. | I would phone the family to inform them of the patient’s condition | □ | □ | □ | □ |
| 10. | I would contact the Chaplain urgently | □ | □ | □ | □ |
| 11. | I would request the cannula is re-sited | □ | □ | □ | □ |
| 12. | I would request a peripherally inserted central catheter is inserted | □ | □ | □ | □ |
| 13. | I would not increase the oxygen without prescription | □ | □ | □ | □ |
| 14. | I would want IV fluids recommenced | □ | □ | □ | □ |
| 15. | I would want IV fluids continuing as before | □ | □ | □ | □ |
| 16. | I would want Mrs F moved out of a general ward environment to a higher dependency setting | □ | □ | □ | □ |

**Part 3**

**Situation – 23.45.** Mrs F is found by night staff; she has pulled her oxygen off and appears clammy and confused, trying to climb out of bed. The FY1 has been bleeped but they are with another patient who is about to arrest on another ward and will come as soon as possible.

**Background.** Abdominal pain caused by renal mass. AF. Referred to Palliative Care Team. Condition has deteriorated over the afternoon and evening**.**

**Assessment.** Respiratory Rate 9, Heart Rate 145, irregular and weak, Blood Pressure 60/30mmHg.o2 saturations unrecordable. She has not passed urine in the past 3 hours.

What would your **Recommendations** be**?**

**Click on the link to see the form (if randomised to the UFTO or DNACPR order). To get back to the scenario click the ‘back arrow’ at the top left corner of the page.**

| **INTERVENTIONS.** | | | | | |
| --- | --- | --- | --- | --- | --- |
|  | | Strongly  agree | Agree | Disagree | Strongly disagree |
| 1. | Observations should be stopped | □ | □ | □ | □ |
| 2. | I would discontinue the fluid balance chart | □ | □ | □ | □ |
| 3. | I would urgently bleep the Registrar | □ | □ | □ | □ |
| 4. | I would not put out a crash call | □ | □ | □ | □ |
| 5. | I would phone the family to inform them of Mrs F’s condition | □ | □ | □ | □ |
| 6. | I would contact the out of hours Chaplain | □ | □ | □ | □ |
| 7. | I would discontinue the Oxygen if it is making her more agitated | □ | □ | □ | □ |
| 8. | I would discontinue IV fluids if the cannula infiltrates again | □ | □ | □ | □ |
| 9. | I would not administer the night time antibiotics | □ | □ | □ | □ |
| 10. | Bag-Valve-Mask ventilations should be started if her respiratory rate decreases below 8 | □ | □ | □ | □ |
| 11. | If Mrs F becomes pulseless I would start CPR | □ | □ | □ | □ |
| 12. | I would not want to start chest compressions if Mrs F has a cardiac arrest | □ | □ | □ | □ |
| 13. | Mrs F should be defibrillated if she goes into a cardiac arrest in a shockable rhythm | □ | □ | □ | □ |
| 14. | I would want Mrs F moved to Intensive Care. | □ | □ | □ | □ |
